# Chinese herbal medicine granules (PTQX) for children with moderate to severe atopic eczema: study protocol for a randomised controlled trial

**DOI:** 10.1186/s13063-015-0806-y

**Published:** 2015-07-07

**Authors:** Sherman X. Gu, Anthony L. Zhang, Meaghan E. Coyle, Xiumei Mo, George B. Lenon, Noel E. Cranswick, DaCan Chen, Charlie C. Xue

**Affiliations:** Traditional and Complementary Medicine Research Program, Health Innovations Research Institute and School of Health Sciences, Royal Melbourne Institute of Technology (RMIT) University, Plenty Road, P.O. Box 71, Bundoora, 3083 VIC Australia; Department of Dermatology, Guangdong Provincial Hospital of Chinese Medicine, 111 Dade Road, Guangzhou, 510120 Guangdong China; Royal Children’s Hospital, Murdoch Children’s Research Institute, University of Melbourne, 50 Flemington Road, Parkville, 3052 VIC Australia; The Guangdong Provincial Academy of Chinese Medical Sciences, 111 Dade Road, Guangzhou, 510120 Guangdong China

**Keywords:** Atopic dermatitis, Atopic eczema, Chinese herbal medicine, Clinical trial, PTQX, RCT

## Abstract

**Background:**

Atopic eczema or atopic dermatitis is a chronic inflammatory skin disease. Current conventional medical treatment for moderate and severe atopic eczema is not satisfactory. There is promising evidence derived from randomised clinical trials to support the clinical use of Chinese herbal medicine in the management of atopic eczema. However, the available evidence is compromised by the high risk of bias associated with most of the included trials. Therefore, well-designed and adequately powered randomised clinical trials are needed. The primary aim of this trial is to evaluate the efficacy and safety of oral ingestion of an oral Chinese herbal formula (Pei Tu Qing Xin granules; PTQX) in children aged between 6 and 16 years with moderate to severe atopic eczema.

**Methods/Design:**

We have designed a randomised, double-blind, placebo-controlled, two-arm, parallel clinical trial with 12 weeks of treatment and a 4-week follow-up period. A pilot study with 30 participants will be conducted at the RMIT University in Australia to determine the feasibility of the full-scale randomised clinical trial (*N* = 124). Eczema Area and Severity Index score will be the primary outcome. Secondary outcome measures include change in symptoms using the Patient-Oriented Eczema Measure, the Children’s Dermatology Life Quality Index and the use of concomitant medicines. Safety parameters include report of adverse events and pathology tests during the trial period.

**Discussion:**

Key elements for conducting a high-quality randomised clinical trial have been addressed in this protocol. Findings from the proposed trial will provide critical evidence regarding Chinese herbal medicine treatment for atopic eczema.

**Trial registration:**

Australian New Zealand Clinical Trials Registry Identifier: ACTRN12614001172695. Date of Registration: 7 November 2014.

## Background

Atopic eczema (AE) or atopic dermatitis is an inflammatory skin disease characterised by redness of the skin, scaling, swelling, accentuation of the hair follicles and lichenification that result from chronic scratching owing to severe itchiness [1]. The prevalence of AE worldwide ranges from 1 % to 20 %. Nigeria, the United Kingdom and New Zealand have the highest reported prevalences [2]. A recent Australian prospective cohort study done over the course of 40 years showed that childhood eczema was strongly associated with the incidence and persistence of adult atopic asthma, suggesting that early treatment may reduce morbidity later in life [3].

Management of mild AE includes topical application of corticosteroids and emollients, and refractory moderate or severe AE is managed with systemic therapies, including oral steroids or immunosuppressive drugs such as azathioprine, cyclosporine, mycophenolate and methotrexate [4, 5]. Current conventional medical treatment for moderate and severe AE is not satisfactory, as long-term oral corticosteroid use can lead to adverse reactions such as suppression of the hypothalamic–pituitary–adrenal axis, osteoporosis, aseptic necrosis of the hip, hypertension, ocular changes and altered immune function [6]. Side effects of immunosuppressive medications, in particular the risk of hepatosplenic T-cell lymphomas associated with azathioprine, have raised alert [7]. Therefore, many people with AE may choose to use Chinese herbal medicine (CHM) for the treatment of AE [8].

CHM has a long history of use, dating back to the second century ad. Unlike conventional medicine, CHM uses a composite of different botanical resources such as barks, seeds, flowers, roots or mineral substances to create a formula which is administered as decoctions, pills, washes, lotions or ointments. There has been increasing interest in evaluating the efficacy and safety of CHM for AE. To determine the benefit of CHM for AE, we completed a Cochrane systematic review of CHM for AE in 2013 [9]. A total of 28 studies were included in the review (2306 participants). There is evidence that CHM has increasingly been used for the management of AE since the publication of the first Cochrane systematic review in this area in 2004. All included trials have presented the benefit of CHM was superior or equivalent to its comparator. However, the promising effect claimed by the trials’ investigators is compromised by the high risk of bias within most of the included trials, and we further suggested that well-designed, adequately powered randomised clinical trials (RCTs) are needed.

### Trial objective

The primary objective of the present trial is to determine the efficacy and safety of oral ingestion of an existing oral Chinese herbal formula (Pei Tu Qing Xin granules; PTQX) on symptom severity and improvement in health-related quality of life (QoL) in children aged between 6 and 16 years with moderate to severe AE. We will also assess the safety of PTQX for AE.

## Methods/Design

**Fig. 1:**
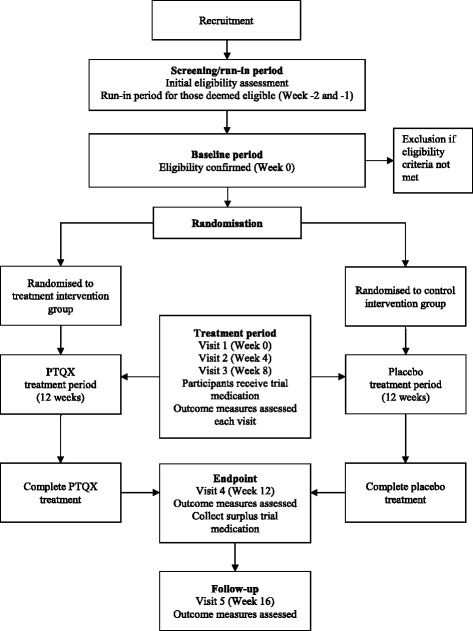
Flowchart of the trial process

The trial will be a 16-week, randomised, double-blind, placebo-controlled, two-arm, parallel clinical trial. The 16-week trial includes 12 weeks of treatment with either PTQX or placebo and a 4-week follow-up period. Figure 1 outlines the process of the trial. A pilot study (*N* = 30) will be conducted to inform a planned larger RCT (*N* = 124). The study protocol was approved by the Royal Melbourne Institute of Technology (RMIT) University Human Research Ethics Committee (HREC), filed with the Therapeutic Goods Administration (TGA) under the Clinical Trial Notification (CTN) scheme (CTN file number: 2014/033590) and registered with the Australian and New Zealand Clinical Trials Registry (ANZCTR) (registration ID: ACTRN12614001172695; dated 7 November 2014).

### Randomisation

A block randomisation method will be employed with balanced variable blocks. Equal numbers of participants will receive the real treatment and the inert vehicle (the placebo). Randomisation codes will be generated by a computer program run by an independent statistician who is not directly involved with the trial.

### Blinding and allocation concealment

Neither the trial participants nor investigators will know the participant allocation to receive real treatment or placebo. The randomisation codes will be concealed in individual opaque envelopes. Envelopes will be sequentially numbered and opened only after the participant’s unique identifier is written on the envelope. At the time of randomization (visit 1, week 0), each participant will randomly pick one sealed envelope from among the group. The envelope will be opened by a CHM dispenser, who will be a registered CHM practitioner or registered CHM dispenser. The trial medication (both PTQX and placebo) will be prepackaged, and the appearance of the packages will be identical. The randomisation code will not be revealed to participants and personnel who are involved in data entry and data analysis. An emergency unblinding envelope with identical contents will be prepared in case a serious adverse event occurs during the treatment period. In case of a serious adverse event where immediate cessation of trial medication is required, the emergency unblinding envelope will be opened for further investigation of the nature of the event by the trial investigators.

### Preparation and administration of the trial medications

The trial medications will be prepared by Jiangyin Tianjiang Pharmaceutical Co. Ltd.*,* a Good Manufacturing Practices–certified CHM extract granules manufacturer. Quality control and quality assurance, such as identification of products and quality and safety testing, were conducted by the manufacturer. The raw herbs were mixed, cooked twice with water, filtered, concentrated, spray-dried and mixed with sucralose and pearl powder (Margarita powder) and finally forming granules (PTQX). They were packaged in small sachets weighing 2 g each. A sample batch of PTQX was tested for microorganisms and is deposited with the manufacturer for traceability. In addition, a fingerprint profile of PTQX has been established by using an ultra high-performance liquid chromatography–high resolution mass spectrometry operated in multistage mode profiling method for further study of the active compounds of the formula.

PTQX is modified from a CHM formula, Qing Xin Pei Tu, which is based on the principle of treatment in strengthening the spleen and eliminating the heart fire in Chinese medicine, and most children with AE manifested with deficiency of the spleen and heart fire [10, 11]. The formula has been used in children with AE for many years [10–12]. The formula is prepared as CHM granules with extracts from nine Chinese medicinal substances, including Rhizoma Atractylodis macrocephalae (Bai Zhu), Radix Pseudostellariae (Tai Zi Shen), Rhizoma Dioscoreae (Shan Yao), Semen Coicis (Yi Yi Ren), Rhizoma Imperatae (Bai Mao Gen), Fructus Forsythiae (Lian Qiao), Radix Glycyrrhizae (Gan Cao), Cortex Dictamni (Bai Xian Pi) and Margarita (Zhen Zhu Feng). In CHM theory, Bai Zhu has the function of strengthening the spleen, and Lian Qiao can clear away the heart fire and remove toxins. These two herbs serve as sovereign (jun) herbs. Bai Mao Gen is used to cool the blood and reduce swelling by promoting diuresis. Tai Zi Shen, Yi Yi Ren and Shan Yao have the function of strengthening the spleen and eliminating dampness. These four substances act as minister (chen) herbs to further enhance the effects of the sovereign herbs. Zhen Zhu Feng calms the mind and extinguishes the wind. Bai Xian Pi has the function of clearing away heat, drying the dampness, expelling the wind and easing itchiness. These two ingredients work as assistant (zuo) role in the formula. Can Cao is a guide (shi) herb, as it harmonises all ingredients of the formula [13]. Evidence derived from preclinical research have shown that Atractylodis macrocephalae and Semen Coicis could improve the immune function by increasing interleukin (IL)-1 or IL-2, and Fructus Forsythiae and Radix Glycyrrhizae have anti-inflammatory action [14].

Four grams of trial medications twice daily (total of 8 g) for 6–7-year-old subjects and 6 g twice daily (total of 12 g) for 8–16-year-old participants will be given to both study groups with written and oral instructions on use of the trial medications.

### Selection and withdrawal of participants

Participants will be recruited through advertisements in local papers, school newsletters, posters, via the internet and through referrals from general practitioners (GPs) or dermatologists. Trial information flyers will be placed in offices of GPs or dermatologists who agree to refer patients to the trial. Contact details of the potential participants who express interest in the trial will be recorded. The *participant information and consent form* with information about the trial, including the purpose of the trial, details of the intervention, and potential adverse reactions, will be given to potential participants. Potential participants will be interviewed to assess their eligibility against the inclusion and exclusion criteria to select suitable participants for the trial (screening and run-in period).

#### Selection criteria

The U.K. Working Party’s diagnostic criteria and the Nottingham Eczema Severity Score (NESS) will be adopted as inclusion criteria for this trial [15–17]. Eligible participants will be boys or girls aged 6–16 years with a history of skin pruritus within the previous 12 months plus three or more of the following: (1) onset under the age of 2 years, (2) history of flexural involvement, (3) history of generally dry skin, (4) personal history of other atopic disease such as asthma or (5) visible flexural dermatitis. Further, eligible participants will have a total NESS score ≥9 (moderate to severe) at baseline and will have provided written informed consent.

Those who used corticosteroids, other immunosuppressives or any preparation of oral herbal medicines for treatment of AE in the past 30 days or who have been diagnosed with scabies, allergic contact dermatitis, seborrhoeic dermatitis or psoriasis will be excluded. Those who have severe medical conditions, such as cardiovascular, liver or renal dysfunction, and/or a history of lactose intolerance will not be included.

#### Withdrawal, dropout and discontinuation

Participants are free to withdraw at any time during the trial. Participants who wish to withdraw will be offered the option to cease trial medication but continue attending scheduled visits for outcome measurement. Participants who withdraw will be followed to investigate the reason for withdrawal. Participants may be advised to discontinue the treatment if there is a product-related adverse event of a serious nature or if the participant was not compliant with the study requirements (e.g., use of oral corticosteroids during the treatment period). Topical corticosteroid use is allowed for those participants who were using topical corticosteroids before trial participation and whose skin conditions worsen during the trial treatment period. These concomitant medications will be documented. Discontinuers will not be replaced by new participants. Intention-to-treat analysis will be performed on missing data from discontinuers with the last observation carried forward method.

### Sample size

Calculation of the sample size of this trial was based on the effect size of the primary outcome, Eczema Area and Severity Index (EASI) score, reported in a previous published study [18]. The means and standard deviations (SDs) of the EASI scores were 7.4 ± 3.3 and 9.6 ± 4.1 for the active treatment and control groups, respectively. To achieve 90 % power (β = 0.10), significance level of 0.05 (α) in a two-tailed test, calculated by using G*Power 3.1.2 (http://www.gpower.hhu.de/en.html), 62 participants, based on an estimated 10 % dropout rate, are needed for each group. That makes a total of 124 for the main trial. A pilot study with 30 participants (15 per group) conducted in RMIT University will provide preliminary data on the clinical efficacy of PTQX based on EASI score and will allow us to monitor for adverse events. The mean and SD of EASI score from the pilot study will be used for recalculation of the sample size for the main trial.

### Screening and run-in, baseline, treatment periods and endpoint

Preliminary screening assessments will be conducted during the 2 weeks of the run-in period (weeks −2 and −1). Only those participants who have met the inclusion and exclusion criteria will be randomised. EASI score and other baseline outcome assessments will be measured.

The treatment period will involve oral ingestion of the trial medications twice daily for 12 weeks. The trial medications will be supplied to participants at visit 1 (week 0), visit 2 (week 4) and visit 3 (week 8). The endpoint is set at visit 4 (week 12) of the trial period.

### Outcome measures

Current recommendations for outcome measurement in RCTs of AE include clinical signs and symptoms, long-term control of flares and health-related QoL [19]. This trial will adopt these recommendations for its outcome measures. The primary outcome measure is change in clinical signs severity based on EASI score. Secondary outcome measures include change in symptoms using the Patient-Oriented Eczema Measure (POEM) [20], health-related QoL using the Children’s Dermatology Life Quality Index (CDLQI) [21], use of concomitant medicines (number of days used and total dosage for corticosteroids) and adverse events and safety (pathology tests).

The EASI, POEM, CDLQI, concomitant medications and adverse events will be recorded at visit 1 (week 0), visit 2 (week 4), visit 3 (week 8), visit 4 (week 12) and visit 5 (week 16). Pathology tests (full blood examination with eosinophil count and immunoglobulin E, liver and renal function tests) will be performed by a qualified phlebotomist at the screening (week −2) and at the second (week 4) and fourth (week 12) visits. Medical advice from a medical officer will be sought for any serious adverse events or for any serious aggravation of the existing skin condition or abnormal pathology results during the trial period.

Any adverse events will be managed in compliance with the guidelines of the *Note for Guidance on Clinical Safety Data Management: Definitions and Standards for Expedited Reporting (CPMP/ICH/377/95)* issued by TGA [22]. A serious adverse event will be reported to the RMIT HREC within 24 hours and then to the TGA within 7–15 days. Table 1 summarises the schedule of the trial and outlines the time points of outcome measures of the trial.

**Table 1:** Schedule of the trial

Items	Screening/run-in period	Visit 1 (baseline)	Visit 2	Visit 3	Visit 4 (endpoint)	Visit 5 (follow-up)
Week of the trial	−2/−1	0	4	8	12	16
Demographic information-taking	+					
Informed consent form (PICF)	+					
General medical history-taking	+					
Severity of AE for inclusion and exclusion of the participants (NESS)	+					
Blood tests	+		+		+	
Clinical sign of AE (EASI)		+	+	+	+	+
Symptoms (POEM)		+	+	+	+	+
QoL score (CDLQI)		+	+	+	+	+
Distributions of treatment or placebo products		+	+	+		
Daily medical record	+	+	+	+	+	+
Monitor of adverse event		+	+	+	+	+
Compliance check		+	+	+	+	+
Survey of participation						+

*Abbreviations: AE* atopic eczema, *CDLQI* Children’s Dermatology Life Quality Index, *EASI* Eczema Area and Severity Index, *NESS* Nottingham Eczema Severity Score, *PICF* participant information and consent form, *POEM* Patient-Oriented Eczema Measure, QoL quality of life

### Statistical analysis

Data will be analysed by an independent statistician using IBM SPSS version 22 software (IBM, Armonk, NY, USA). Comparison of means of continuous variables (i.e., EASI, POEM and CDLQI scores) will be expressed as differences in means with 95 % confidence intervals (CIs). One-way analysis of variance will be used for normally distributed data, and nonparametric tests will be used for skewed distribution data. χ^2^ or Fisher’s exact tests will be performed for comparison of dichotomised data, and dichotomous data will be expressed as risk ratios with 95 % CIs. All comparisons will be two-tailed, with *p* values <0.05 considered to be statistically significant.

## Discussion

Determination of the trial timeline is one of the challenges of the trial design. It depends on many factors, such as how long the effect of the intervention may last, costs of the trial and tolerability by and compliance of the participants. The trial duration of 16 weeks has been determined after consideration of these issues. This protocol was developed according to the guidelines of *Australian Clinical Trial Handbook* [23] and follows the *nine CONSORT checklist items for RCTs of herbal medicines* [24]. Key elements for conducting a high-quality RCT, such as randomisation, sequence generation, allocation concealment, blinding methods, proper sample size, compliance with protocol, determination of baseline and endpoint and adequate outcome measures have been addressed.

## Trial status

Participant recruitment commenced on 31 March 2015. It is anticipated that the trial will be completed in February 2016.

## Abbreviations

AE: Atopic eczema
ANZCTR: Australian New Zealand Clinical Trials Registry
CDLQI: Children’s Dermatology Life Quality Index
CHM: Chinese herbal medicine
CI: Confidence interval
CONSORT: Consolidated Standards of Reporting Trials
CTN: Clinical Trial Notification
EASI: Eczema Area and Severity Index
GP: General practitioner
HREC: Human Research Ethics Committee
IL: Interleukin
NESS: Nottingham Eczema Severity Score
PICF: Participant information and consent form
POEM: Patient-Oriented Eczema Measure
PTQX: Pei Tu Qing Xin granules
QoL: Quality of life
RCT: Randomised clinical trial
RMIT: Royal Melbourne Institute of Technology
SD: Standard deviation
TGA: Therapeutic Goods Administration
